# Contractility defects hinder glycoprotein VI-mediated platelet activation and affect platelet functions beyond clot contraction

**DOI:** 10.1016/j.rpth.2024.102322

**Published:** 2024-01-22

**Authors:** Martin Kenny, Alice Y. Pollitt, Smita Patil, Dishon W. Hiebner, Albert Smolenski, Natalija Lakic, Robert Fisher, Reema Alsufyani, Sebastian Lickert, Viola Vogel, Ingmar Schoen

**Affiliations:** 1 School of Pharmacy and Biomolecular Sciences, Royal College of Surgeons in Ireland, Dublin, Ireland; 2 Irish Centre for Vascular Biology, Royal College of Surgeons in Ireland, Dublin, Ireland; 3 School of Biological Sciences, University of Reading, Reading, United Kingdom; 4 School of Medicine, Conway Institute, University College Dublin, Belfield, Dublin, Ireland; 5 School of Medicine, Royal College of Surgeons in Ireland, Dublin, Ireland; 6 Department of Health Sciences and Technologies, ETH Zurich, Zurich, Switzerland

**Keywords:** blebbistatin, hemostasis, nonmuscle myosin type IIA, thrombosis, traction

## Abstract

**Background:**

Active and passive biomechanical properties of platelets contribute substantially to thrombus formation. Actomyosin contractility drives clot contraction required for stabilizing the hemostatic plug. Impaired contractility results in bleeding but is difficult to detect using platelet function tests.

**Objectives:**

To determine how diminished myosin activity affects platelet functions, including and beyond clot contraction.

**Methods:**

Using the myosin IIA-specific pharmacologic inhibitor blebbistatin, we modulated myosin activity in platelets from healthy donors and systematically characterized platelet responses at various levels of inhibition by interrogating distinct platelet functions at each stage of thrombus formation using a range of complementary assays.

**Results:**

Partial myosin IIA inhibition neither affected platelet von Willebrand factor interactions under arterial shear nor platelet spreading and cytoskeletal rearrangements on fibrinogen. However, it impacted stress fiber formation and the nanoarchitecture of cell-matrix adhesions, drastically reducing and limiting traction forces. Higher blebbistatin concentrations impaired platelet adhesion under flow, altered mechanosensing at lamellipodia edges, and eliminated traction forces without affecting platelet spreading, α-granule secretion, or procoagulant platelet formation. Unexpectedly, myosin IIA inhibition reduced calcium influx, dense granule secretion, and platelet aggregation downstream of glycoprotein (GP)VI and limited the redistribution of GPVI on the cell membrane, whereas aggregation induced by adenosine diphosphate or arachidonic acid was unaffected.

**Conclusion:**

Our findings highlight the importance of both active contractile and passive crosslinking roles of myosin IIA in the platelet cytoskeleton. They support the hypothesis that highly contractile platelets are needed for hemostasis and further suggest a supportive role for myosin IIA in GPVI signaling.

## Essentials

• Platelet-driven clot contraction is important to form a stable plug after blood vessel injury.
• Myosin IIA drives clot contraction but is not known for other roles in platelets.
• A small reduction in myosin IIA activity has severe consequences for clot contraction.
• Complete loss of myosin IIA activity also makes platelets less responsive to collagen.

## 1 Introduction

Platelets perform a multitude of tasks during thrombus formation, from initial rolling to firm adhesion, granule secretion, aggregation, clot contraction, and provision of a procoagulant surface. Several of these steps are mechanical in nature and affected by external fluid shear forces [1]. The extent to which contractile forces generated within the platelet cytoskeleton affect these diverse platelet functions is, in contrast, poorly understood but has implications for diagnosing platelet function defects and developing safer antiplatelet drugs. Recent findings show that impaired platelet contractility is (1) causative for bleeding in *MYH9*-related disease (*MYH9*-RD) [2], a hereditary platelet disorder with mutations in the *MYH9* gene coding for the nonmuscle myosin IIA heavy chain; (2) prevalent in patients with other cytoskeletal defects or bleeding of unknown cause [3]; (3) a hallmark for the action of aspirin and P2Y_12_ inhibitors *in vitro* and in cardiology patients [4]; and (4) indicative of bleeding risk in patients with trauma [4]. While reduced platelet contractility might arise as the final consequence of different conditions, it is intimately linked with reduced or impaired myosin activity. Nonmuscle myosin IIA is a hexamer composed of 2 heavy chains (Myh9), 2 essential light chains, and 2 regulatory light chains (RLCs). Phosphorylation of the RLC at Thr18/Ser19 leads to the adoption of an activated unfolded conformation, which, if not phosphorylated at its heavy chain [5], allows myosin monomers to assemble into bipolar filaments [6] to unbind from acidic lipids in the plasma membrane [7] and hydrolyze adenosine triphosphate (ATP). ATP hydrolysis enables f-actin binding, thereby establishing physical crosslinks in the cytoskeleton, while continued energy consumption drives motor stepping, sliding of actin filaments, and generation of contractile forces. Impaired myosin activity can be studied experimentally using mouse models [8] or pharmacologic inhibitors. Selective inhibition of myosin IIA ATPase activity by blebbistatin (BBT) [9] abolishes clot contraction [4,10] and results in thrombus instability, with frequent embolization events and rebleeding [10,11], analogous to observations with *MYH9*-RD models [2]. Since responses to soluble agonists are largely preserved in *MYH9*-RD platelets, including integrin α_IIb_β_3_ activation and α-granule secretion [2] as well as largely normal aggregometry results apart from minor defects in platelet shape change [12,13], the answer to why platelet contractility is required for hemostasis has mainly focused on clot stability as the principle mechanism. However, myosin also coregulates the assembly of the actin cytoskeleton, which is involved in fundamental processes such as spreading [11], focal adhesion maturation [14], and substrate stiffness sensing [[15], [16], [17], [18]], which are largely preserved across cell types, as well as platelet granule secretion [19]. This raises the possibility that myosin functionality might also be important for earlier steps in thrombus formation. Given the unique role of myosin IIA in force generation and the correlation between bleeding tendency and contractility defects [3,4,20], a more comprehensive understanding of the underlying biophysical mechanisms in single platelets is warranted.

In this study, we selectively modulated myosin IIA activity in human platelets from healthy donors using BBT to avoid confounding effects from altered expression levels or macrothrombocytopenia in *MYH9*-RD platelets [2,8] and comprehensively investigated the resulting functional changes at various levels of inhibition. The bundling of actin filaments into stress fibers and the nanoscale architecture of focal adhesions were most sensitive to partial myosin inhibition, effectively eliminating the formation of highly contractile platelets. More severe myosin inhibition impacted all mechanosensitive biomechanical platelet functions, whereas solution-based aggregation and secretion assays remained largely insensitive. Unexpectedly, we discovered that myosin activity contributes to downstream glycoprotein (GP)VI signaling. These data show that fully active myosin IIA is required for efficient force generation in platelets and suggest that the role of myosin extends to platelet functions beyond clot contraction.

## 2 Methods

For material sources and additional methods, see the Supplementary Methods section in the Supplementary Information.

### 2.1 Blood collection and platelet preparation

This research was approved by the Royal College of Surgeons in Ireland Research Ethics Committee (#1394, #1504). Procedures followed local legislation and the Declaration of Helsinki. Blood was collected from healthy volunteers into citrate blood collection tubes after obtaining informed consent. Washed platelets or platelet-rich plasma were prepared as usual [21].

### 2.2 Traction force microscopy

Elastomeric micropost arrays (mPADs) were replica-molded, functionalized with Alexa Fluor 488-labeled fibrinogen, and passivated with DyLight 405-labeled bovine serum albumin [22]. Washed platelets were seeded in Tyrode’s 2-[4-(2-hydroxyethyl)piperazin-1-yl]ethanesulfonic acid (HEPES) buffer containing 1.8 mM CaCl_2_, 5 μM adenosine diphosphate (ADP), and BBT or vehicle (dimethyl sulfoxide [DMSO], 0.2% final concentration) on mPADs at 37 °C, fixed after 60 minutes, stained with Alexa Fluor 647-phalloidin, and imaged on an inverted epifluorescence microscope (X73, Olympus) using a 100×/1.4 Numerical Aperture (NA) UPlanSApo objective and filter sets for 4′,6-diamidino-2-phenylindole, fluorescein, and Cy5 on an EM-CCD camera (iXon Ultra 888, Andor Technology). Image stacks (129-nm pixel size, 0.2-μm z-steps) were deconvolved (Huygens Deconvolution, Scientific Volume Imaging), and the bending of posts was determined using custom image analysis code (Matlab version 2021a, MathWorks) [22].

### 2.3 Dynamic platelet function assay

Flow chamber experiments on von Willebrand factor (VWF) coatings were performed [21] at a wall shear rate of 1500 s^−1^ and imaged at 50 fps over 30 seconds. Platelets were localized and tracked [23,24]. Exponential fits of the number of platelets over time, $n\left(t\right)=k_{on}/k_{eff}\left(1-\exp\;\left(-\left(t+3s\right)k_{eff}\right)\right)$, yielded the adhesion rate $k_{on}$ and the equilibrium coverage $n_{\infty }=k_{on}/k_{eff}$. In agreement with previous studies, the proportion of platelets traveling less than 3.5 μm was defined as fraction static [24,25]. Histograms of velocity and distances of all platelet traces were fitted by a single exponential.

### 2.4 Microscopy of spread platelets

Washed platelets (1.3 × 10^6^ cm^−2^) were seeded on fibrinogen-coated coverslips for 60 minutes or on collagen-coated coverslips for 10 minutes in Tyrode’s HEPES buffer with 1.8 mM CaCl_2_ at 37 °C, washed, fixed in 3% paraformaldehyde for 15 minutes, and permeabilized. For morphometrics, samples were stained for vinculin (Alexa Fluor 546) and with Alexa Fluor 488-phalloidin, mounted in Mowiol, and imaged on an Examiner Z1 confocal microscope (Zeiss) using a 40×/1.3 NA oil objective lens, laser excitation at 488 nm and 546 nm, and 70-nm pixel size. Single-cell features were measured using an automated morphometric analysis [26]. For lifetime stimulated emission depletion (τSTED), samples were stained for myosin or vinculin (Alexa Fluor 594) and with Star635P-phalloidin, mounted in Mowiol, and imaged on a Stellaris τSTED (Leica Microsystems) using a 100×/1.40 NA oil objective, laser excitation at 590 nm and 640 nm, 30% of a 785-nm depletion laser in a 2D donut, and 20-nm pixel size. Images were visualized and analyzed in Fiji [27] and using published Matlab scripts [26]. For stochastic optical reconstruction microscopy (STORM), samples were stained for vinculin or GPVI (Alexa Fluor 647) and with Alexa Fluor 488-phalloidin, postfixed in 2% paraformaldehyde for 10 minutes, and imaged in photoswitching buffer on a homemade microscope setup using a 63×/1.45 NA oil immersion objective, laser excitation at 640 nm, increasing 405 nm activation laser power to maintain a constant number of blinking events per frame, and 108-nm pixel size [28]. Localization, visualization, and analysis were performed using SMAP [29].

### 2.5 Platelet aggregation

Washed platelets (4 × 10^5^ μL^−1^ in Tyrode’s HEPES buffer with 1.8 mM CaCl_2_ and 0.5 mg/mL fibrinogen) were preincubated with para-amino BBT or an equivalent DMSO concentration for 5 minutes at 37 °C. The BBT derivative para-amino BBT was used to avoid phototoxicity [30]; similar results were obtained with BBT. Blanking was performed using the relevant BBT concentration (Supplementary Figure S1). Light transmission was monitored by a PAP-8 aggregometer (Bio/Data Corp).

### 2.6 Immunoreceptor tyrosine-based activation motif signaling

Procedures were approved by the University of Reading Research Ethics Committee. Washed platelets were resuspended at 8 × 10^5^ μL^−1^ and rested for 30 minutes at 30 °C. Experiments were performed under nonaggregating conditions in the presence of 9 μM eptifibatide (α_IIb_β_3_ inhibitor), 10 μM indomethacin (cyclooxygenase inhibitor), and 2 U/mL apyrase (ATP scavenger). Following 10 minutes of incubation with para-amino BBT, platelets were stimulated with crosslinked collagen–related peptide (CRP-XL) under stirring (1200 rpm) for 90 seconds before lysis in 1X sodium dodecyl-sulfate sample buffer for analysis by sodium dodecyl-sulfate polyacrylamide gel electrophoresis and Western blotting using phospho-specific or total protein antibodies. Fluorophore-conjugated primary or secondary antibodies were visualized using a Typhoon FLA 9500 (GE Healthcare), and band intensities were quantified using Image Quant software (GE Healthcare).

### 2.7 Calcium signaling

Platelet-rich plasma was incubated with 1 μM Cal-520-AM at 30 °C for 60 minutes and then centrifuged at 300 ×*g* for 20 minutes. Platelets were resuspended at 5 × 10^5^ μL^−1^ in Tyrode’s HEPES buffer with 1.8 mM CaCl_2_ and rested in the presence of secondary inhibitors (9 μM eptifibatide, 30 μM acetylsalicylic acid [cyclooxygenase inhibitor], and 0.5 U/mL apyrase) for 30 minutes. Platelets (100 μL) were incubated with inhibitors in a 96-well black transparent-bottom plate at 37 °C for 10 minutes. Fluorescence emission was excited at 480/40 nm and recorded at 525/50 nm every 10 seconds on a Fluoroskan Ascent FL (Thermo Fisher Scientific). CRP-XL (2 μg/mL) was added at 60 seconds for 360 seconds. Intracellular calcium concentration was calculated as [Ca^2+^]_i_ = k_d_ (F_max_ − F)/(F − F_min_), with k_d_ = 320 nM for Cal-520 and F = the measured fluorescence intensity. F_max_ was determined after lysing platelets using 1% Triton X-100, shaking, and 10 minutes of incubation. F_min_ was subsequently determined analogously after adding 20 mM ethylene glycol-bis(β-aminoethyl ether)-*N,N,N,N′*-tetraacetic acid.

### 2.8 ATP secretion

Dense granule release was measured using an ATP luminescence multiplate format assay [31]. Washed platelets (3.5 × 10^5^ μL^−1^ in Tyrode’s HEPES buffer with 1.8 mM CaCl_2_) were preincubated with para-amino BBT or 0.08% (v/v) DMSO and secondary inhibitors (10 μM ARC69931 [P2Y_12_ antagonist], 100 μM MRS2179 [P2Y_1_ antagonist], 5 μM acetylsalicylic acid, and 9 μM eptifibatide) for 3 minutes at 37 °C, added to a white 96-well plate (Sigma Aldrich), and activated with 0.5 μg/mL CRP-XL for 3 minutes on a Victor2 1420 multilabel counter (Perkin Elmer) under fast orbital shaking. Chrono-Lume reagent diluted 1:10 (v/v) was added, luminescence was read, and values were background-subtracted and referenced to the luminescence intensity of 40 nM ATP calibrator solutions, including the relevant para-amino BBT concentrations.

### 2.9 Statistical analysis

Plots of data were prepared and statistical analysis of data was performed in GraphPad Prism (version 10.0.0, GraphPad Software) using one-way analysis of variance with Bonferroni correction for multiple comparisons. Significance levels were set at .05. Additional data visualization and fitting were performed in Matlab (version 2022a, MathWorks).

## 3 Results

### 3.1 Platelet traction forces are highly sensitive to partial myosin IIA inhibition by BBT

**Figure 1:**
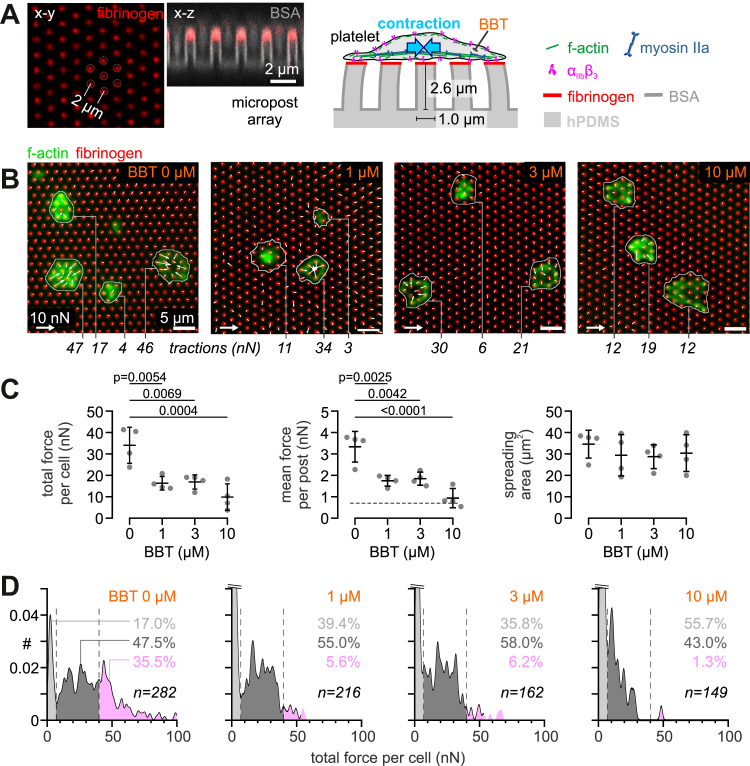
Platelet traction forces are highly sensitive to partial myosin IIA inhibition by blebbistatin (BBT). (A) Assay format for traction force microscopy. Elastomeric polydimethylsiloxane (PDMS) micropost arrays were microcontact-printed with fibrinogen at their tops and passivated with bovine serum albumin (BSA) elsewhere. A single platelet specifically adheres to, spreads over, and pulls at several microposts, as mediated by α_IIb_β_3_ integrins and the actomyosin cytoskeleton. Scale bars: 2 μm. (B) Fluorescence images of f-actin (green) of fixed washed human platelets on fibrinogen-functionalized micropost arrays (red) after spreading for 60 minutes in the absence/presence of subsaturating concentrations of BBT. Indicated are total single-cell traction forces. Scale bars: 5 μm. (C) Total traction force per cell (left), mean traction force per post (middle), and spreading area (right) of washed platelets on micropost arrays at subsaturating concentrations of BBT. The dashed line indicates the measurement force resolution of individual posts. Mean values and SD from 4 independent biological replicates with 24 to 94 cells per condition are shown. Statistical tests were performed using one-way analysis of variance with Bonferroni correction for multiple comparisons; only *P* values of <.05 are indicated. (D) Frequency histograms of total traction forces per platelet at subsaturating concentrations of BBT. Histograms were subdivided into noncontractile cells (<7 nN; light gray) and cells with medium (7-40 nN; dark gray) or high (>40 nN; pink) contractility. The frequency of subpopulations with different levels of contractility is indicated; note that the peak of noncontractile cells was cropped to maintain a consistent y-axis scaling. Data were pooled from 4 independent biological replicates; the total number of cells is indicated as *n*.

Reduced platelet contractility is a hallmark of *MYH9*-RD [2,3]. We determined whether BBT concentrations around its half maximal inhibitory concentration of ∼5.1 μM for inhibiting human myosin IIA ATPase activity could replicate key features of this condition. To this end, we investigated platelets from healthy donors at 0, 1, 3, and 10 μM BBT using traction force microscopy on fibrinogen-coated elastomeric mPADs (Figure 1A). Platelets adhered to, spread, and pulled on the microposts under all conditions (Figure 1B and Supplementary Figure S2A). While platelet spreading area was unaffected by BBT, the total force exerted by single platelets was halved already at the lowest tested BBT concentration (Figure 1C). The mean force per post covered by a single platelet decreased accordingly but remained above the measurement sensitivity for 1 and 3 μM BBT, while it was diminished further by 10 μM BBT.

Since highly contractile platelets have been proposed to be important for hemostasis [3], we further investigated the heterogeneity of platelet tractions (Figure 1D). The percentage of highly contractile platelets (>40 nN total force per platelet) dropped from 35.5% for the control to 5.6% for 1 μM BBT, a dramatic relative reduction by ∼85%. Concomitantly, the fraction of platelets that were spread but did not exert any traction forces increased 2.3-fold from 17.0% to 39.4%. Thus, we conclude that BBT at low concentrations primarily affected highly contractile platelets rather than the ability of platelets to develop intermediate levels of contractility.

### 3.2 Partial myosin inhibition reduces the assembly of force-bearing cell-matrix junctions and force-generating stress fibers

**Figure 2:**
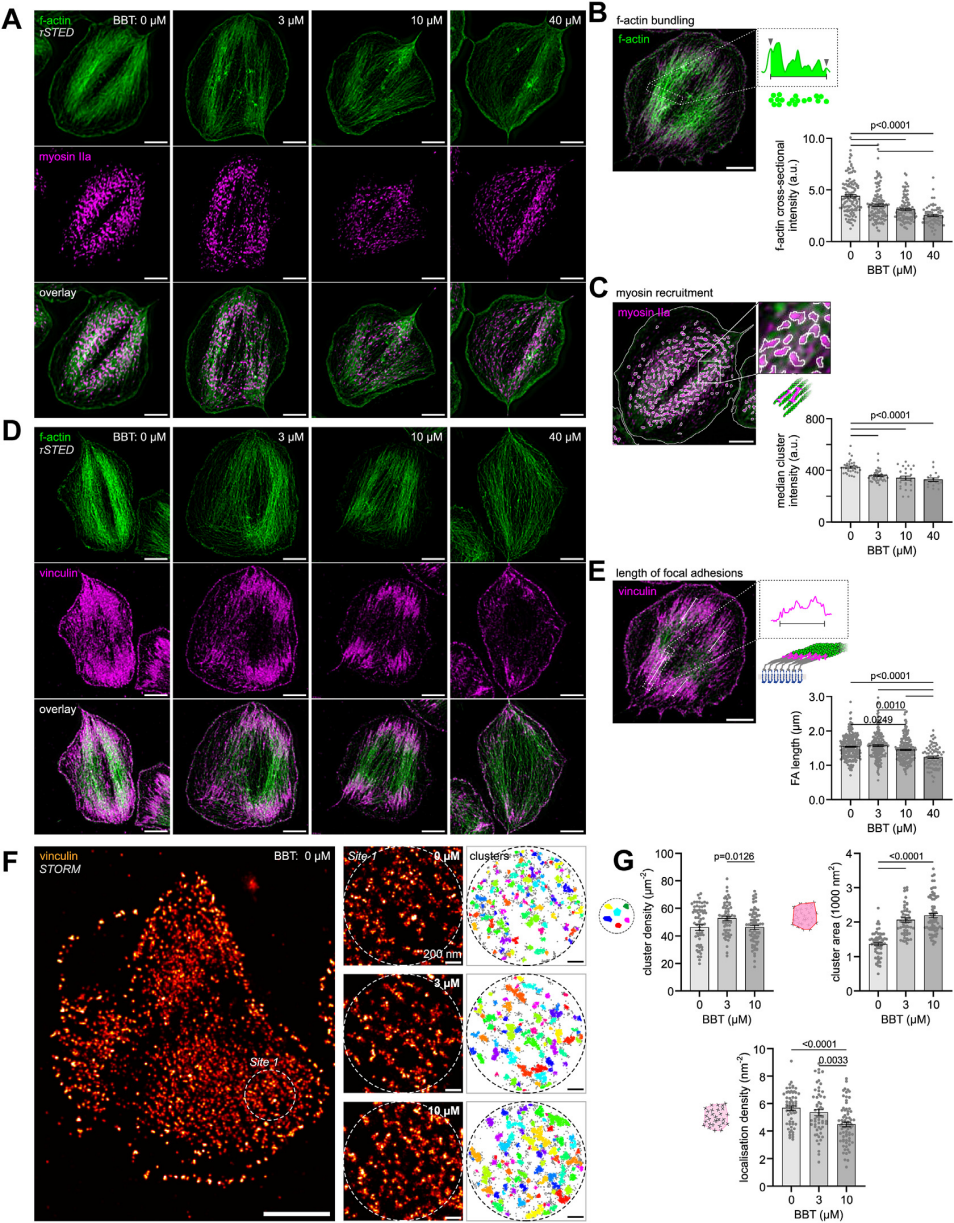
Partial myosin IIA inhibition reduces the assembly of force-bearing cell-matrix junctions and force-generating stress fibers. (A) Dual-color lifetime stimulated emission depletion (τSTED) super-resolution images of f-actin (green) and myosin IIA (magenta) in representative platelets at the indicated blebbistatin (BBT) concentrations. For images of additional platelets, see Supplementary Figure S3A. Scale bars: 2 μm. (B) Analysis of actin filament bundling within actin stress fibers as measured by the cumulative f-actin intensity per width determined from perpendicular line profiles. Shown are data with mean ± SEM from 64 to 138 line profiles in 25 to 58 cells per experimental condition. Scale bar: 2 μm. (C) Analysis of myosin recruitment into stress fibers as measured by the median intensity of clusters detected by thresholding of the myosin staining. Shown are data with mean ± SEM from 17 to 43 cells per experimental condition. Scale bar: 2 μm. (D) Dual-color τSTED super-resolution images of f-actin (green) and vinculin (magenta) in representative platelets at the indicated BBT concentrations. For images of additional platelets, see Supplementary Figure S3B. Scale bar: 2 μm. (E) Analysis of focal adhesion length from the vinculin staining. Shown are data with mean ± SEM from 80 to 286 focal adhesions in 22 to 49 cells per experimental condition. Scale bar: 2 μm. (F) Vinculin clustering in stochastic optical reconstruction microscopy (STORM) super-resolution data. Sites of interest in focal adhesions (left) were selected and subjected to a density-based spatial clustering of applications with noise (DBSCAN) analysis with a neighborhood radius of 17 nm and minimum points of 15 (right). Scale bars: 2 μm (main), 200 nm (sites). (G) Analysis of vinculin clustering. Shown are the density of clusters in a focal adhesion (top left), the mean area of a cluster (top right), and the mean density of localizations within clusters (bottom) with data with mean ± SEM from 57 to 71 sites in 21 to 26 cells per experimental condition. All statistical tests were performed using one-way analysis of variance with Bonferroni correction for multiple comparisons; only *P* values of <.05 are indicated.

We next determined how partial pharmacologic inhibition of myosin ATPase activity affected the buildup of mechanosensitive complexes (Figure 2). Actin stress fibers are major contractile structures that control focal adhesion assembly in nucleated cells [32]. When spread on immobilized fibrinogen, platelets develop stress fiber-like f-actin bundles that span across the cell and are anchored in vinculin-rich focal adhesion sites. However, the detailed structure of these functional units is typically not clearly discernible due to the diffraction-limited resolution of confocal microscopy. We used τSTED microscopy to resolve and analyze f-actin organization in more detail (Figure 2A and Supplementary Figure S3A). The density of actin filaments within stress fiber-like bundles was significantly decreased by 3 μM BBT and even further at higher concentrations (Figure 2B). The looser organization of actin filaments coincided with an apparently sparser recruitment of myosin IIA into f-actin bundles (Figure 2A). Quantification of individual myosin clusters showed a drop in intensity at 3 μM BBT (Figure 2C), which potentially indicates that fewer myosins assembled into bipolar filaments, similar to observations made for high concentrations of BBT in nucleated cells using electron microscopy [33,34]. While the length of focal adhesions, which is a measure of their maturation status [14,32], was barely affected by low-dose BBT (Figure 2D, E and Supplementary Figure S3B), vinculin was significantly less tightly clustered at 3 μM BBT compared with control, as evidenced by a larger cluster area and fewer localizations per cluster in STORM data (Figure 2F, G). In summary, reduced myosin IIA ATPase activity resulted in a weakening of force-bearing platelet-matrix adhesion complexes and force-generating actin stress fibers.

### 3.3 Partial myosin IIA inhibition preserves mechanotransduction by primary adhesion receptors

**Figure 3:**
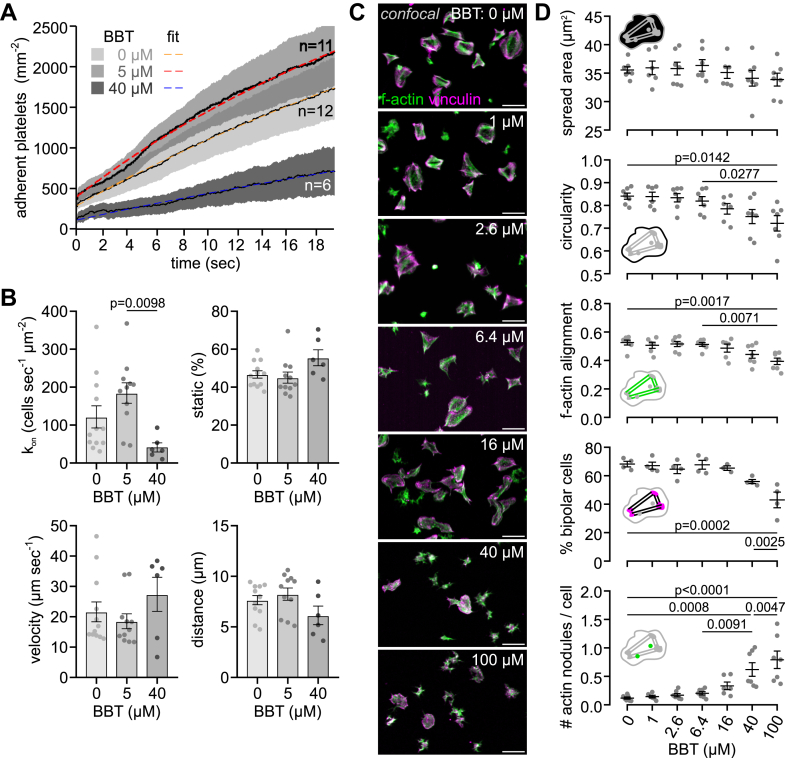
Partial myosin IIA inhibition preserves mechanotransduction by primary adhesion receptors. (A) Effect of para-amino blebbistatin (BBT) on glycoprotein Ib-mediated adhesion of platelets to von Willebrand factor (VWF) under arterial shear (1500 s^−1^). Whole blood was preincubated with vehicle, 5 or 40 μM para-amino BBT, and 3,3′-dihexyloxacarbocyanine iodide (DiOC_6_) for 5 minutes at 37 °C and then perfused through flow channels with VWF-coated glass bottoms. Movies were analyzed for the number of adherent platelets over time. Shown are the mean ± SEM of 6 to 12 independent experiments (Supplementary Figure S4). Exponential fits yielded equilibrium coverages (95% CIs) of 4556 (4497-4615), 4233 (4160-4306), and 2545 (2352-2737) cells mm^−2^ for 0, 5, and 40 μM para-amino BBT, respectively. (B) Adhesion rate k_on_, fraction static, mean translocation velocity, and mean translocation distance of platelets adhering and rolling on VWF. Data were obtained by analysis of the same movies as in A. Error bars depict mean ± SEM. (C) Representative confocal images of platelets on fibrinogen-coated glass after 60 minutes of spreading at various BBT concentrations under static conditions. Green: f-actin; magenta: vinculin. Scale bars: 10 μm. (D) Confocal analysis of single-cell morphology in terms of (top to bottom) spreading area, circularity, f-actin alignment, the percentage of bipolar cells, and the average number of actin nodules per platelet. Mean values from 4 to 7 independent biological replicates with 262 to 564 cells per condition are shown. Error bars depict mean ± SEM. All statistical tests were performed using one-way analysis of variance with Bonferroni correction for multiple comparisons; only *P* values of <.05 are indicated.

The initial contact of platelets with injured blood vessel walls is mediated by mechanosensitive interactions between the GPIb-IX complex and VWF [1]. Since GPIb becomes linked by filamin to the actin cytoskeleton, we asked whether internal actomyosin pulling forces might affect this process, analogous to the role of external shear forces. Based on the comparison of the accumulation (Figure 3A) and translocation behavior (Figure 3B) of single platelets on VWF under arterial shear in a flow chamber assay [23,24], no significant differences were found between no or low-dose (5 μM) BBT. High-dose (40 μM) BBT caused a significant reduction of the adhesion rate and resulted in tendentially less platelet interactions with the surface, but faster rolling and shorter distance traveled; however, these did not reach statistical significance due to the relatively large variability between individual experiments (Supplementary Figure S4). We conclude that elevated platelet contractility is not required for arresting platelets on VWF under flow, but a complete loss of myosin functionality reduces the ability of platelets to attach under shear.

The development of traction forces depends on the maturation of initial cell-matrix contacts and the reorganization of the platelet cytoskeleton [35,36] mediated by mechanosensing through integrins and the actomyosin machinery [18]. We next assessed the effect of BBT on the morphology of platelets spread on fibrinogen-coated glass under static conditions using a high-throughput assay [26]. In accordance with platelets on microposts (Figure 1C), the adhesion (Supplementary Figure S2B) and spreading area on flat glass were unaffected by BBT up to 100 μM (Figure 3C, D). BBT concentrations greater than 16 μM led to more irregular cell shapes, reduced the reorganization of f-actin bundles and focal adhesions into an aligned, bipolar morphology, and led to a significant increase in the frequency of actin nodules (Figure 3D). However, no significant changes in these characteristics were detected at lower BBT concentrations, indicating that a low level of myosin activity is sufficient for substrate sensing, spreading, and cytoskeletal rearrangements.

### 3.4 The role of myosin IIA contractility in platelet aggregate formation depends on the activation pathway

**Figure 4:**
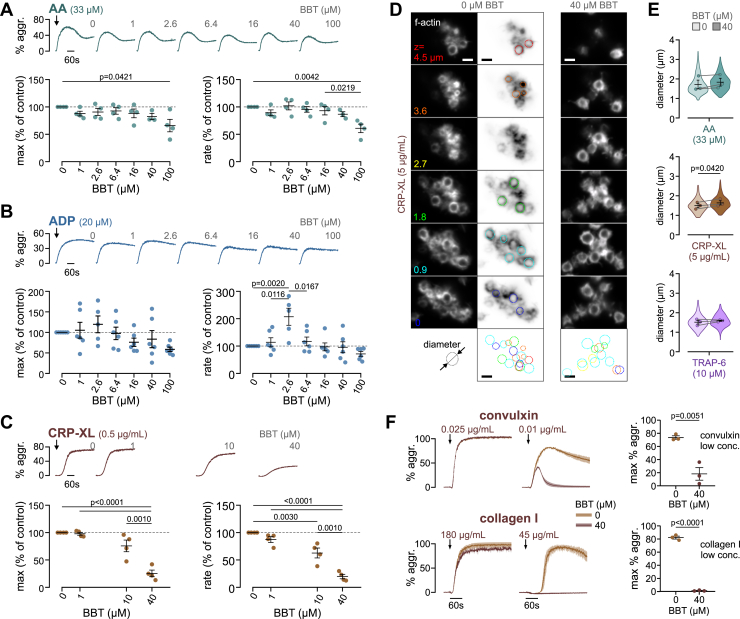
The role of myosin IIA contractility in platelet aggregate formation depends on the activation pathway. Light transmission aggregometry of washed platelets preincubated with different para-amino blebbistatin (BBT) concentrations and stimulated by (A) arachidonic acid (AA), (B) adenosine diphosphate (ADP), or (C) crosslinked collagen-related peptide (CRP-XL). Shown are representative aggregation curves (top), maximum aggregation (left), and rate of aggregation (right) of 4 to 6 independent experiments. Values are shown normalized to control (0 μM BBT). Maximum aggregation in controls was (mean ± SD) 42.5 ± 5.3% for AA, 40.0 ± 6.2% for ADP, and 68.0 ± 6.8% for CRP-XL. Error bars depict the mean ± SEM. All statistical tests were performed using one-way analysis of variance with Bonferroni correction for multiple comparisons; only *P* values of <.05 are indicated. (D) Aggregates formed from washed platelets were fixed when reaching 30% aggregation, stained for f-actin, and imaged by confocal microscopy. Z-stacks of example aggregates formed using 5 μg/mL CRP-XL at 0 μM or 40 μM BBT. The diameter of platelets in the aggregate was determined by manual measurements from confocal slices (dashed-colored outlines). Scale bars: 2 μm. (E) Violin plots and means ± SEM of average platelet diameters per experimental replicate (points) from aggregates formed by AA (top), CRP-XL (middle), or thrombin receptor activating peptide-6 (TRAP-6; bottom) in the absence/presence of 40 μM BBT. Violin plots comprise 47 to 224 platelets from 12 to 16 aggregates from 3 experimental replicates per condition. Statistical comparisons were performed using a paired Student’s *t*-test. (F) Aggregation curves of washed platelets stimulated with saturating (left) or subsaturating (middle) concentrations of convulxin (top) or collagen type I (bottom). Curves show the mean and range of 2 technical replicates representative of 3 independent experiments. Right: maximum percent aggregation at low agonist concentrations for 3 independent experiments. Statistical comparisons were performed using an unpaired Student’s *t*-test.

The formation of a thrombus requires integrin α_IIb_β_3_ activation that enables fibrin(ogen)-mediated platelet-platelet interactions and clot contraction. To test the sensitivity of these processes on myosin activity, we induced aggregation of washed platelets under stirring conditions in a light transmission aggregometer by the addition of different agonists (Figure 4). Arachidonic acid–induced platelet aggregation was unaltered by BBT, except for a ∼40% reduction at the highest concentration of 100 μM BBT (Figure 4A). A similar, although more variable, response was obtained using 20 μM ADP, except for a +20% higher and significantly 2-fold faster aggregation at the intermittent 2.6 μM BBT concentration (Figure 4B). In contrast, platelet aggregation induced by CRP-XL, which activates platelets through GPVI, was significantly inhibited and slowed down by 10 to 40 μM BBT by as much as 80% (Figure 4C).

Since light transmission aggregometer does not provide any information on the structure or properties of platelet aggregates, these were further investigated by confocal microscopy. Aggregates formed in solution contained rounded platelets with mainly cortical f-actin staining (Figure 4D). The diameter of single platelets within aggregates was measured as a surrogate marker for their contraction. In comparison with resting platelets which had a diameter of 3.16 ± 0.46 μm (Supplementary Figure S5), platelets in aggregates formed by arachidonic acid, CRP-XL, or a protease-activated receptor-1 (PAR1) agonist (thrombin receptor activating peptide-6) had significantly reduced sizes of 1.72 ± 0.31 μm, 1.49 ± 0.14 μm, and 1.51 ± 0.13 μm, respectively (Figure 4E), because of shape change and contraction. BBT partially inhibited the contraction of platelets induced by CRP-XL (+0.15 μm) but not significantly when arachidonic acid (+0.11 μm) or thrombin receptor activating peptide-6 (+0.08 μm) was used. Notably, a higher concentration of CRP-XL was used in these experiments to overcome the inhibition of aggregate formation by BBT (cf Figure 4C).

We next asked if the sensitivity to BBT was a general feature of GPVI-stimulated platelet aggregation since GPVI activation mechanism may differ depending on agonist type [37]. BBT had no or only a minor effect at high concentrations of the snake venom convulxin or type I collagen but significantly reduced or completely abolished responses at subsaturating agonist concentrations (Figure 4F).

In summary, platelet aggregate formation and contraction induced by soluble activators was largely independent of myosin activity, whereas (submaximal) stimulation through GPVI, which under physiological conditions occurs at the subendothelium, was sensitive to myosin inhibition.

### 3.5 Myosin IIA activity contributes to GPVI downstream signaling

Before exploring possible mechanisms through which myosin inhibition interfered with GPVI-stimulated platelet responses, we verified that BBT did not change platelet size, GPVI surface expression levels, or GPVI shedding in resting and convulxin-stimulated suspended platelets (Supplementary Figure S6).

**Figure 5:**
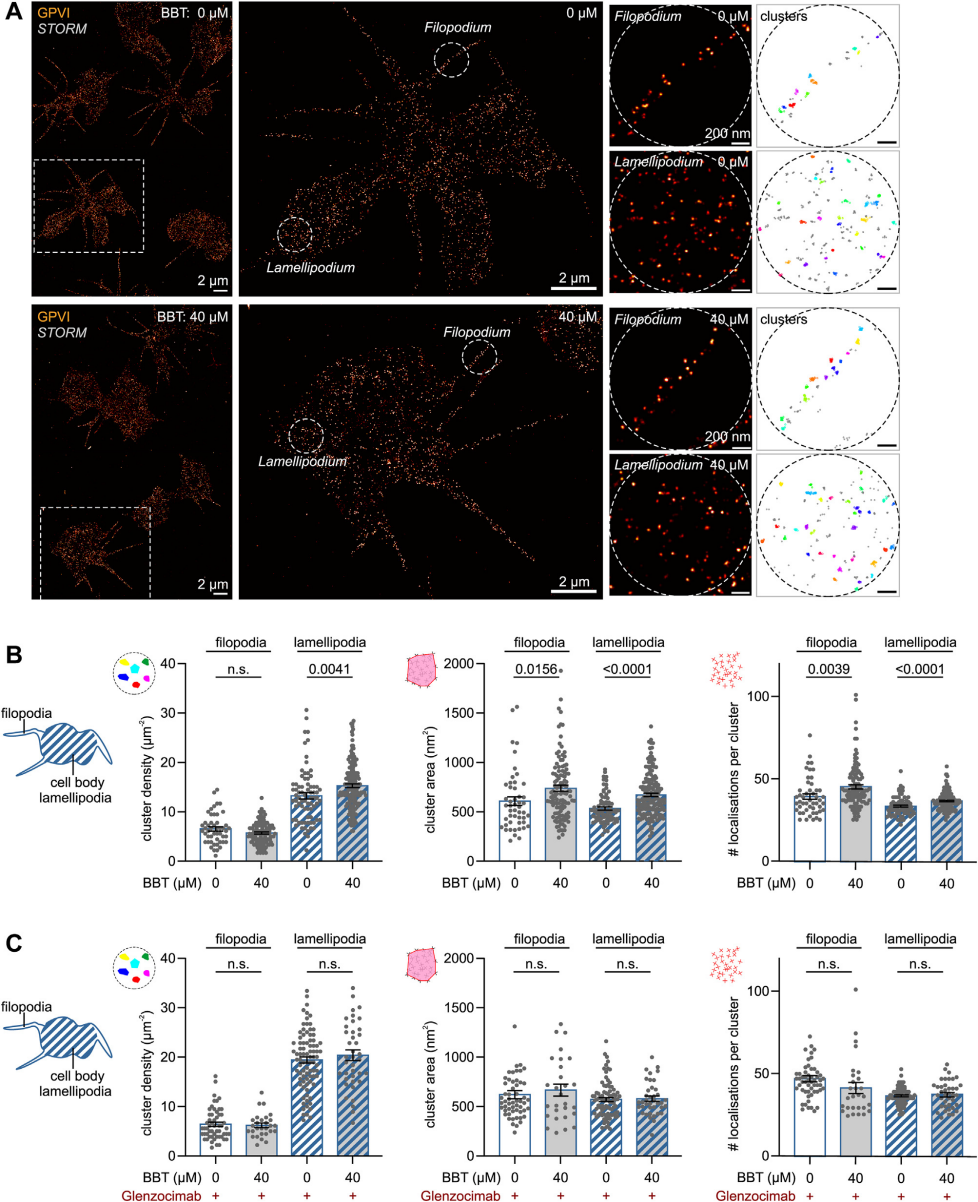
Myosin IIA activity affects glycoprotein (GP)VI clustering and surface rearrangements. Washed platelets were seeded for 5 minutes onto collagen-coated coverslips in the presence of 40 μM para-amino blebbistatin (BBT) or an equivalent concentration of dimethylsulfoxide (DMSO), fixed, and immunolabeled for GPVI. (A) GPVI clustering in stochastic optical reconstruction microscopy (STORM) super-resolution data. Sites of interest in filopodia or lamellipodia were selected (middle) and subjected to a density-based spatial clustering of applications with noise (DBSCAN) analysis with a neighborhood radius of 30 nm and minimum points of 10 (right). Scale bars: 2 μm (main), 200 nm (sites). (B) Analysis of GPVI clustering. Shown are the density of clusters (left), the mean area of clusters (middle), and the mean number of localizations within clusters (right) obtained from 50 to 160 sites in 33 to 37 cells per experimental condition. Shown are individual data points with mean ± SEM of 2 biological replicates. Open bars: sites in filopodia; striped bars: sites in lamellipodia/cell body. Statistical comparisons were performed using an unpaired Student’s *t*-test; only *P* values of <.05 are indicated. (C) Experiments were performed in the presence of (and after preincubation with) the GPVI antagonist glenzocimab (40 μg/mL). STORM analysis was performed as above. Shown are the density of clusters (left), the mean area of clusters (middle), and the mean number of localizations within clusters (right) obtained from 28 to 86 sites in 14 to 21 cells per experimental condition. Shown are individual data points with mean ± SEM. Open bars: sites in filopodia; striped bars: sites in lamellipodia/cell body. Statistical comparisons were performed using an unpaired Student’s *t*-test; only *P* values of <.05 are indicated. N.s., not significant.

Accompanying the initiation of GPVI signaling, glycine-proline-hydroxyproline repeat motif-containing ligands like CRP-XL or fibrillar collagens induce the clustering of GPVI-FcRγ complexes and their rearrangement at the membrane surface, which can be measured using STORM [38]. We thus investigated if BBT affected GPVI clustering in spreading platelets 5 minutes after first contact with a collagen-coated surface. STORM images and density-based spatial clustering of applications with noise (DBSCAN) analysis detected GPVI clusters in both lamellipodia and filopodia (Figure 5A). Platelets incubated with 40 μM BBT showed significantly higher cluster densities in lamellipodia with larger cluster areas and more localizations per cluster than vehicle control (Figure 5B). Similar yet less significant differences were observed for GPVI clusters located in filopodia. Blocking of GPVI signaling by glenzocimab blunted all differences in GPVI clustering (Figure 5C). Of note, the number of clusters per area in lamellipodia was about 30% higher in the presence of glenzocimab (cf Figure 5B, C), indicating that myosin inhibition still allows for initial GPVI clustering but affects secondary GPVI clustering or surface rearrangements downstream of initial GPVI signaling.

**Figure 6:**
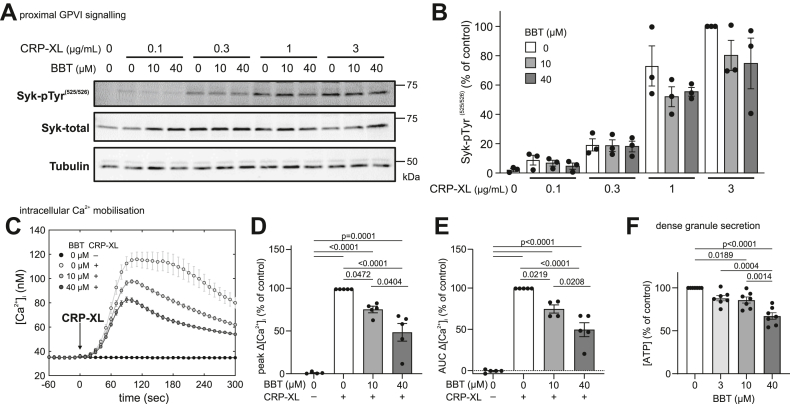
Myosin IIA activity contributes to glycoprotein (GP)VI downstream signaling. Washed platelets in solution were stimulated using crosslinked collagen–related peptide (CRP-XL) under nonaggregating conditions in the presence of secondary inhibitors (see Methods). (A) Representative Western blot of total and tyrosine phosphorylated Syk in the presence of 0, 10, or 40 μM para-amino blebbistatin (BBT) 90 seconds after stimulation with 0, 0.1, 0.3, 1, or 3 μg/mL CRP-XL. (B) Quantification of Syk tyrosine phosphorylation from Western blots. Shown are individual data points with mean ± SEM of 3 independent biological replicates. (C) Intracellular calcium [Ca^2+^]_i_ was measured in response to 3 μg/mL CRP-XL in the presence of 0, 10, and 40 μM para-amino BBT. Shown are [Ca^2+^]_i_ over time of a representative experiment (left; mean and SD of triplicates). Differences in (D) peak Ca^2+^ concentration or (E) cumulative Ca^2+^ influx (area under the curve [AUC]) over 300 seconds after stimulation. Responses were normalized to control (peak: mean [range], 96.4 [29.3-169.63] nM; AUC: mean [range], 18,497 [5134-35,526] nM sec) to limit donor-to-donor and experimental variability. Shown are individual data points with mean ± SEM of 5 biological replicates. (F) Adenosine triphosphate (ATP) release was measured in response to 0.5 μg/mL CRP-XL in the presence of 0, 3, 10, and 40 μM para-amino BBT. ATP concentrations were normalized to control (mean [range], 15.3 [0.7-68.6] nM) to limit donor-to-donor variability. Shown are individual data points with mean ± SEM of 7 biological replicates. All statistical tests were performed using one-way analysis of variance with Bonferroni correction for multiple comparisons; only *P* values of <.05 are indicated.

GPVI-FcRγ clustering recruits Src family kinases to phosphorylate FcRγ’s immunoreceptor tyrosine-based activation motifs (ITAMs), activating Syk and the PLCγ2 pathway to result in intracellular Ca^2+^ elevation, dense granule secretion, and integrin α_IIb_β_3_ activation [39]. We thus next tested whether proximal and/or downstream GPVI signaling were sensitive to myosin IIA inhibition. Increasing concentrations of CRP-XL led to increasing total tyrosine phosphorylation (Supplementary Figure S7A) and increasing Syk-pTyr^(525/526)^ phosphorylation (Figure 6A) in suspended platelets 90 seconds after stimulation, which was tendentially (by 20%-30%) but not significantly reduced by BBT (Figure 6B). While the initial 90-second rising phase of intracellular Ca^2+^ mobilization was unaffected by BBT, Ca^2+^ peak concentration and cumulative influx over 5 minutes were dose-dependently and significantly reduced by up to ∼55% (Figure 6C–E), as was Syk phosphorylation after 210 seconds (Supplementary Figure S7B). Furthermore, BBT dose-dependently reduced GPVI-mediated dense granule secretion 3 minutes after stimulation by up to 33% (Figure 6F). GPVI signaling is further known to activate Rho GTPases responsible for the coordination of cytoskeletal rearrangements underlying the development of contractile stress fibers (through RhoA) or the secretion of α-granules [40,41]. Accordingly, CRP-XL stimulation induced a 3-fold increase of active RhoA (Supplementary Figure S8) and robust P-selectin expression (Supplementary Figure S9); however, these responses were unaffected by BBT. GPVI stimulation further contributes to the formation of procoagulant platelets if the cytosolic Ca^2+^ increase is prolonged by concurrent PAR or integrin signaling [42]. Accordingly, CRP-XL robustly triggered phosphatidylserine (PS) exposure of thrombin costimulated platelets in solution (Supplementary Figure S10A) or of spread platelets on immobilized CRP-XL (Supplementary Figure S10B). However, PS exposure was unaffected by myosin inhibition. Previous experiments, in contrast, had observed an increased PS exposure and more frequent ballooning in the presence of 80 μM BBT [43], potentially in part due to the higher BBT dose used or the phototoxicity of BBT during live cell experiments [30,44]. Consistent with our own findings, thrombin generation aided by CRP-XL prestimulated platelets was insensitive to the presence of BBT (Supplementary Figure S10C).

In summary, myosin inhibition significantly reduced Ca^2+^ mobilization, dense granule secretion, and GPVI clustering downstream of GPVI but had a minor effect on proximal GPVI ITAM signaling during the early phase and a negligible effect on α-granule secretion or procoagulant platelet formation.

## 4 Discussion

We here show that graded myosin IIA inhibition causes a spectrum of nonphysiological changes in platelets that highlight the diverse roles of myosin IIA for clot contraction and beyond under physiological and pathologic conditions.

Our findings can be interpreted in accordance with the regulation of myosin IIA activity and the known pharmacologic effects of BBT. Although BBT’s half maximal inhibitory concentration is measured by inhibition of ATP hydrolysis [9,30], BBT rather blocks phosphate release [45,46], which abolishes motor stepping while still allowing weak f-actin binding, whereas higher BBT concentrations also prevent the formation or promote the disassembly of bipolar myosin filaments [34,47]. Accordingly, we observed a progressive loosening of actin filament bundling and reduced myosin recruitment into the cytoskeleton of platelets at low BBT concentrations, which altered the nanoscale architecture of force-bearing adhesion complexes (Figure 2). Most importantly, blocking ∼50% of myosin’s ATP activity left enough motors of bipolar filaments intact to generate intermediate-level traction forces (Figure 1), analogous to the dose-dependent effect of BBT on fibroblasts on stiff microposts [48] and on actin filament sliding over immobilized myosins [9]. Only fully functional myosin IIA yielded highly contractile platelets, which could be explained by the presence of binding-competent but stepping-incompetent motors in BBT-treated bipolar filaments that disturb the collective stepping motion of motor assemblies [49] and thus impair the efficiency of actomyosin contraction. Since the same low BBT concentrations completely abolished thrombus contraction but not thrombus buildup [4], these findings together suggest that a subpopulation of highly contractile platelets is indispensable for clot stabilization, supporting a hypothesis originally put forward based on the absence of highly contractile platelets in certain patients with bleeding [3].

GPIb-IX signals are transduced through 14-3-3 family member proteins similar to PAR receptor signals [50], which lead to Ca^2+^ mobilization and rapid (within 5 seconds) RLC phosphorylation [51]. Although myosin thus very probably becomes activated during platelet rolling on VWF (1-10 seconds), it remains unclear if myosin-mediated f-actin crosslinking or filament sliding develops within this short time frame. In any case, initial GPIb-IX-mediated interactions of platelets with VWF under arterial shear flow were barely affected by BBT (Figure 3A, B), not excluding that internal actomyosin forces could enhance the bond between GPIb and VWF’s A1 domain over longer time scales or under stationary conditions, as suggested previously [52]. Integrin α_IIb_β_3_ outside-in signaling on immobilized fibrinogen was maintained even at the highest BBT concentration, as evidenced by normal spreading (Figure 3C, D). The appearance of membrane ruffles giving rise to irregular cell shapes was a sign of defective mechano-probing at the cell edge [53]. Stress fiber formation, focal adhesion maturation, and traction force development, which normally occur concomitantly over 5 to 20 minutes [45,54,55], were eliminated at high BBT concentrations, as expected from results with fibroblasts [34,48]. Inside-out activation of α_IIb_β_3_ integrins downstream of arachidonic acid and ADP receptors was largely insensitive to myosin inhibition, leaving platelet aggregation in solution intact (Figure 4).

Our findings that GPVI-mediated Ca^2+^ mobilization and consequently dense granule secretion (Figure 6) and platelet aggregation at low stimulus strength (Figure 4) were sensitive to BBT led us to hypothesize that myosin activity might coregulate GPVI downstream signaling and maybe, more generally, even ITAM downstream signaling. Support for this hypothesis comes from a recent report that showed that BBT potently inhibited FcγRIIA-mediated platelet spreading on aggregated immunoglobulin Gs (IgGs) [56] at concentrations where it does not interfere with spreading on fibrinogen (Figure 3), pointing toward an FcγRIIA-specific effect. Moreover, pharmacologic inhibition of myosin or myosin light chain kinase reduced FcR γ-chain phosphorylation, Syk recruitment, and phagocytic cup formation around IgG-opsonized beads in RAW 264.7 macrophages [57]. We only observed a minor reduction in Syk phosphorylation by BBT in CRP-XL-stimulated platelets (Figure 6A, B); however, BBT affected GPVI reorganisation at the platelet membrane downstream of GPVI signaling in spreading platelets on collagen (Figure 5). These congruent findings raise the question through which mechanism myosin might affect GPVI/ITAM downstream signaling. Myosin and actin dissociate from lipid rafts upon GPVI and CLEC-2 stimulation [58], while GPVI-FcRγ translocates to lipid rafts [59,60], together with P2X1 channels of the ATP-gated P2X family [61]. P2X1 channels play an important role in amplifying platelet responses to submaximal GPVI stimuli [62,63]. Since BBT mainly affected platelet responses at low GPVI agonist concentrations (Figure 4, Figure 6) but not strong stimuli (Figure 4F and Supplementary Figures S6, S9, and S10), we hypothesize that myosin IIA plays an active role in the reorganization of membrane receptors involved in signal amplification. A study showing that myosin IIA associates with and facilitates the gating of another P2X family member, P2X7 [64], supports this idea. Further studies are needed to clarify if myosin IIA activity also contributes to P2X1 activity or how else it facilitates signal transduction through GPVI/ITAM receptors in platelets and myeloid cells.

Myosin IIA inhibition by BBT does not mimic altered (pro)platelet formation by megakaryocytes [5] and thus is not a direct model for *MYH9*-RD; however, it is still useful to understand key features of this and other conditions in which upstream regulators of contractility may be affected. Hemostasis-compromising platelet contractility defects are observed on a spectrum ranging from mild to severe. *MYH9*-RD heterozygous mouse models showed residual platelet tractions [2] comparable with platelets treated with low-dose BBT (Figure 1), whereas homozygous mutations comparable with high-dose BBT are embryonically lethal [2,8,65]. Both models have in common that total myosin IIA and RLC expression levels are unaltered, whereas only a fraction is functional (BBT) or phosphorylated (*MYH9*-RD) [2]. The observed reduction in crosslinking of f-actin bundles by low-dose BBT (Figure 2) is associated with a reduction in cell stiffness of adherent fibroblasts [48], and thus, it is probably also relevant for the reduced stiffness of adherent *MYH9*-RD platelets [2]. Most other cellular processes are unaffected in *MYH9*-RD or by low-dose BBT, including GPVI-induced integrin activation and P-selectin expression [2]. Interestingly, *MYH9*-RD platelets exhibited reduced platelet-collagen and platelet-platelet adhesion forces as well as lessened and more unstable thrombus formation on collagen-coated surfaces under flow [2], whereas low-dose BBT did not affect platelet translocation on VWF (Figure 3) or thrombus formation on collagen [4], and thrombus instability was reported only for high-dose BBT [10]. We hypothesize, based on our inhibition data, that reduced phosphorylation of RLC in *MYH9*-RD, which is not replicated by BBT treatment [47], could affect myosin IIA filament assembly and localization more widely through the mechanisms explained above and thus contribute to this difference. Since more than 50% of downstream GPVI-mediated signaling remained intact upon mild myosin inhibition (Figure 6), comparable with patients with heterozygous mutations in GPVI, the reduced GPVI signaling is not expected to cause a bleeding phenotype [60]. Further studies are needed to understand how the upstream regulation of myosin phosphorylation or other signaling pathways affects platelet contractility and, therefore, might contribute to mild bleeding tendencies.

Not having information on the sociocultural background of our platelet donors is not expected to undermine the validity of our findings since our study preselected healthy subjects. A better understanding of how myosin IIA function in platelets is affected by different disease conditions beyond *MYH9*-RD, including those associated with socioeconomic status or inherited traits, holds promise to identify common pathways underlying hemorrhagic tendencies, as well as to develop improved *in vitro* tests to identify patients at risk of bleeding [20]. On the contrary, platelet-driven clot contraction increases the resistance to clot lysis [66] and thus has important implications for the treatment of ischemic stroke. While our study was limited to pharmacologic interventions of myosin IIA activity, and more work will be needed to elucidate underlying mechanisms, our approach and the new results obtained provide a foundation for the planning and interpretation of further studies in *in vivo* models and patients.
