# Supplementary material for: Contractility defects hinder glycoprotein VI-mediated platelet activation and affect platelet functions beyond clot contraction
Source: Res Pract Thromb Haemost. 2024 Jan 22;8(1):102322. doi: 10.1016/j.rpth.2024.102322 (PMC10877441; doi:10.1016/j.rpth.2024.102322)
Supplement: Supplementary Material [file mmc1.pdf]

Supplementary information accompanying the article

Contractility defects hinder GPVI-mediated platelet activation and affect platelet functions beyond clot contraction

Martin Kenny[1,2], Alice Y. Pollitt[3], Smita Patil[1,2], Dishon W. Hiebner[1,2], Albert Smolenski[4], Natalija Lakic[5], Robert Fisher[5], Reema Alsufyani[5], Sebastian Lickert[6], Viola Vogel[6], Ingmar Schoen[1,2]

[1] School of Pharmacy and Biomolecular Sciences, Royal College of Surgeons in Ireland, 123 St. Stephen's Green, Dublin 2, Ireland
[2] Irish Centre for Vascular Biology, Royal College of Surgeons in Ireland, 123 St. Stephen's Green, Dublin 2, Ireland
[3] School of Biological Sciences, University of Reading, Reading, United Kingdom
[4] School of Medicine, Conway Institute, University College Dublin, Belfield, Dublin 4, Ireland
[5] School of Medicine, Royal College of Surgeons in Ireland, 123 St. Stephen's Green, Dublin 2, Ireland
[6] Department of Health Sciences and Technologies, Gloriastrasse 37/39, ETH Zurich, CH-8092 Zurich, Switzerland

Correspondence: Ingmar Schoen, email: ingmarschoen@rcsi.ie, phone: +353 1 402 5062

SUPPLEMENTARY METHODS:

Materials. Adenosine diphosphate disodium salt (#A2754), apyrase from potato (#A6410), ASA (#A5376), bovine serum albumin (#A7906), catalase from bovine liver (#C30), collagen type I from calf skin (#C9791), D(+) glucose (#G8270), fibrinogen from human plasma (#F3879), glucose oxidase type VII (#G2133), mercaptoethanolamine (#M9768), Mouse anti Vinculin antibody (#V9131), and prostaglandin E1 (#P5515) were purchased from Merck. Rabbit anti non-muscle myosin IIA heavy chain (MHCIIA; #909802) was from Biolegend. Alexa Fluor 488 (AF488) phalloidin (#A12379), AF488 NHS ester (#A20000), AF594 NHS ester (#A37572), AF647 phalloidin (#A22287), AnnexinV AF555 (#A35108), 3,3'-dihexyloxacarbocyanine iodide ($DiOC_6$; #D273), Donkey anti Mouse IgG antibody AF546 (#A10036) and DyLight 405 NHS ester (#46400) were from Invitrogen. MRS2179 (#0900) and ARC69931 (#5720) were from Tocris. NF449 (sodium salt; #13324) and JNJ-47965567 (#21895) were from Cayman Chemical. Unconjugated Donkey anti Mouse IgG (#715-005-150) and Donkey anti Rabbit IgG (#715-005-152) antibodies were from Jackson ImmunoResearch. PRP-reagent (#86196), FluCa-kit (#86197) and thrombin calibrator (#86192) were purchased from Stago. Others: Alexa Fluor 647 Mouse Anti-Human Platelet GPVI (#564701; Becton Dickinson); arachidonic acid (#101297; Bio/Data Corp.); (±)-blebbistatin (#203390; Calbiochem), Cal-520 AM (#21130; AAT Bioquest); Chrono-Lume reagent (#395; Chrono-Log Corporation); citrate blood collection tubes (S-Monovette, 10 ml; Sarstedt); collagen type I (101562; BioData Corporation); Convulxin (#5D-67111; Quadratech Diagnostics); CRP-A (Pplus Skin Care Limited); CRP-XL (CambCol Laboratories); eptifibatide (Integrilin; GlaxoSmithKline); flow chambers (#DPFA-1; EFJ Engineering); hPDMS (#PP2-RG07; Gelest); Chrono-Lume reagent (#395; Chronolog Corp); anti β-tubulin Alexa Fluor 488 (#MAS5-16308-A488; Invitrogen); Mouse anti phospho tyrosine (#05-321; Millipore); Mouse anti Syk (#644302; Biolegend); Mouse anti RhoA (sc-418, Santa Cruz Biotechnology); PE-conjugated anti-CD62P antibody (#555524; Becton Dickinson); iFluor555-conjugated anti-CD41 antibody (#10410090-AATB; 2B Scientific); para-amino blebbistatin (#DR-AM-89; Motorpharma Ltd); Rabbit anti MHCIIA antibody (#909802; Biolegend); Rabbit anti phospho Syk (Tyr525/526; #2710; Cell Signaling Technology); RUC-4 (gift from Barry S. Coller); Star635P phalloidin (#ST635P-0100; Abberior); Sylgard 184 (Dowsil); TRAP-6 (#HY-P0078; Insight Biotechnology Ltd); VWF concentrate (Haemate-P; CSL Behring).

STORM imaging. The photoswitching buffer for STORM contained 0.2 M Tris pH 8.2, 10 mM NaCl, 0.5 mg mL^{-1} glucose oxidase, 0.1 mg mL^{-1} catalase, 4% (w/v) D(+) glucose, and 35 mM mercaptoethanolamine (cysteamine) as reducing agent. The imaging laser (640 nm) was set to a power of 4 kW cm^{-2} at the sample plane, and an even illumination intensity over the field of view was achieved by imaging the magnified image of a multimode fibre exit into the sample as described previously [1]. The power of the activation laser (405 nm) was automatically adjusted to maintain a constant number of activations per frame over the duration of the movie. Between 20'000-50'000 frames were acquired at 50 s^{-1}, depending on when the activation ceased. All frames were used for image reconstruction in SMAP [2] to ensure that a comparable number of blinking events per fluorophore is used independent of the sample labelling density. Localisation events spanning adjacent frames were combined; all localisations with a Log-Likelihood ratio better than -1 and a localisation precision better than 15 nm were used for reconstruction and cluster analysis.

RhoA activation. Washed platelets were pre-treated with vehicle or para-amino para-amino BBT for 10 minutes at room temperature and activated with 0.5 µg/mL CRP-XL for 5 minutes at room temperature. Platelet samples treated with para-amino BBT or vehicle but not activated were lysed and RhoA-GTP was precipitated using the Rho-binding domain of Rhotekin fused to GST. Total and precipitated RhoA were analysed by Western blotting.

Flow cytometry. Recalcified washed platelets were pre-treated with vehicle or para-amino BBT for 10 minutes at 37°C, activated with 5 μg/mL CRP-XL for 20 minutes at room temperature, stained with PE-conjugated anti-CD62P antibody (#555524; Becton Dickinson) or isotype control for 10 minutes, then fixed, quenched and diluted before measuring on a flow cytometer (Attune NxT, Invitrogen). Unstained platelet samples were used to gate platelets using side scatter height and forward scatter, and a gate for marker-positive platelets was selected based on the isotype controls. At least 100,000 platelets were analyzed per condition. For GPVI surface level measurements, the protocol was slightly changed by using 5 μg/mL convulxin for 60 minutes for stimulation and using anti-GPVI and anti-CD41 for staining. Gates based on forward and side scatter and for CD41+ cells were used to analyze GPVI signal intensity.

GPVI Shedding. Washed platelets were incubated with either DMSO or 40 μM para-amino BBT for 10 mins at 37°C. Half of the platelet suspension was centrifuged and pellet was dissolved in RIPA buffer for lysis. Other half was diluted 1:1 in Tyrode's buffer and incubated with 0.5 μg/mL convulxin at RT for 60 min. The platelets were pelleted and lysed using RIPA buffer. The samples were reduced using Laemmli SDS sample buffer (6X) (Thermo) and 40-60 μg protein per well was run on SDS PAGE. The proteins were blotted onto PVDF membrane using iBlot™ 2 Gel Transfer Device (Thermo Fisher Scientific). The membrane was blocked for 60 min with 5% BSA in TBST (TBS with 0.5% Tween 20) at RT, incubated with anti-GPVI Alexa Fluor® 647 antibody for 2 hours at RT, washed with TBST and imaged on Amersham Imager 600 using red fluorescence channel. Blots were washed in TBST and probed for GAPDH (Rabbit anti-human GAPDH antibody) using HRP labelled secondary antibody and imaged with ECL detection reagent.

Thrombin generation assay. Experiments were performed at 37°C. PRP containing aggregation inhibitor (9 μM eptifibatide) was preincubated with BBT or DMSO for 10 minutes, stimulated with CRP-XL for 10 minutes under orbital shaking, and pipetted into 96 wells containing PRP reagent or calibrator solutions. FluCa was added and fluorescence was measured every 20 s for 1 h using Thrombinoscope software on microplate fluorometer (Fluoroskan; ThermoScientific).

PS exposure of spread platelets. Washed platelets (1.3 10^6 cm^{-2}) were seeded on CRP-XL-coated coverslips for 45 minutes in Tyrode's-HEPES w/ 1.8 mM $CaCl_2$ at 37°C, then incubated for another 30 minutes in the presence of AnnexinV-AF555 (1:40 in Annexin-V binding buffer: 10 mM HEPES, 2.5 mM $CaCl_2$, 140 mM $NaCl_2$, pH 7.4) at RT, fixed in 3% paraformaldehyde for 15 min, counterstained using $DiOC_6$, and imaged as for morphometry.

SUPPLEMENTARY REFERENCES:

1 Deschamps J, Rowald A, Ries J. Efficient homogeneous illumination and optical sectioning for quantitative single-molecule localization microscopy. *Opt Express* 2016; **24**: 28080.

2 Ries J. SMAP: a modular super-resolution microscopy analysis platform for SMLM data. *Nat Methods* 2020; **17**: 870–2.

SUPPLEMENTARY FIGURES:

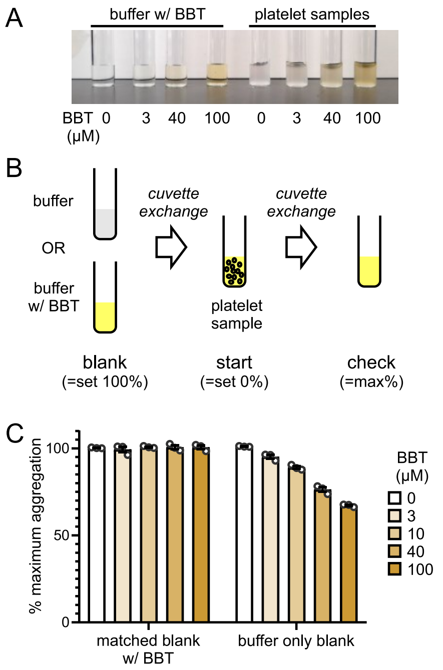

Supplementary Figure S1: BBT absorptivity affects optical LTA measurements and must be included in blanking solutions to obtain valid maximum readings. LTA experiments shown here and in Figure 4 were performed with para-amino BBT.
A) Samples. Washed platelets in Tyrode's buffer were supplemented with different para-amino BBT concentrations. Solutions containing the same BBT concentrations but no platelets were used as controls.
B) Test strategy. Blanking was performed either with the para-amino BBT solution matching the para-amino BBT concentration in the platelet sample, or with buffer only, and maximum aggregation readings were determined by using these 'clear' solutions containing para-amino BBT.
C) Results. The maximum achievable % aggregation was 100% when using matched blanking solutions containing para-amino BBT but was reduced to ~67% at 100 µM para-amino BBT concentration.
When using BBT instead of para-amino BBT for LTA, qualitatively similar but more drastic effects were observed, with a reduction of the % maximum aggregation to only~45% at 100 µM BBT without proper blanking (data not shown). Similar results were also obtained when using platelet-poor plasma as a 'buffer' in the case that LTA is performed with PRP instead of washed platelets (data not shown).

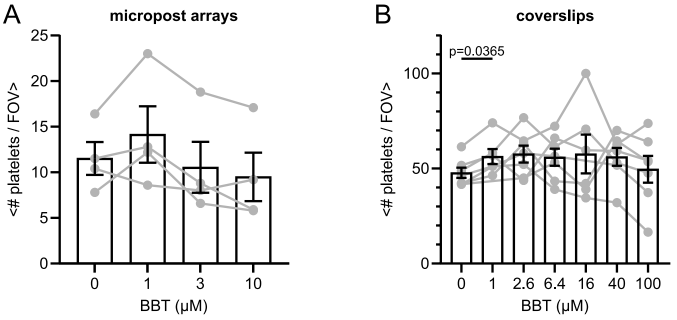

Supplementary Figure S2: Platelet adhesion under stationary conditions on A) fibrinogen-coated micropost arrays (n=4) or on B) fibrinogen-coated glass (n=7) is not affected by different concentrations of BBT. Shown are the average number of platelets per field of view (FOV) from 4-8 images per condition and biological replicate. Note that the FOV for B) was about 5x larger than for A). Statistical comparisons were performed using one-way ANOVA with Bonferroni correction for multiple comparisons. Only differences with $p<0.05$ significance are shown.

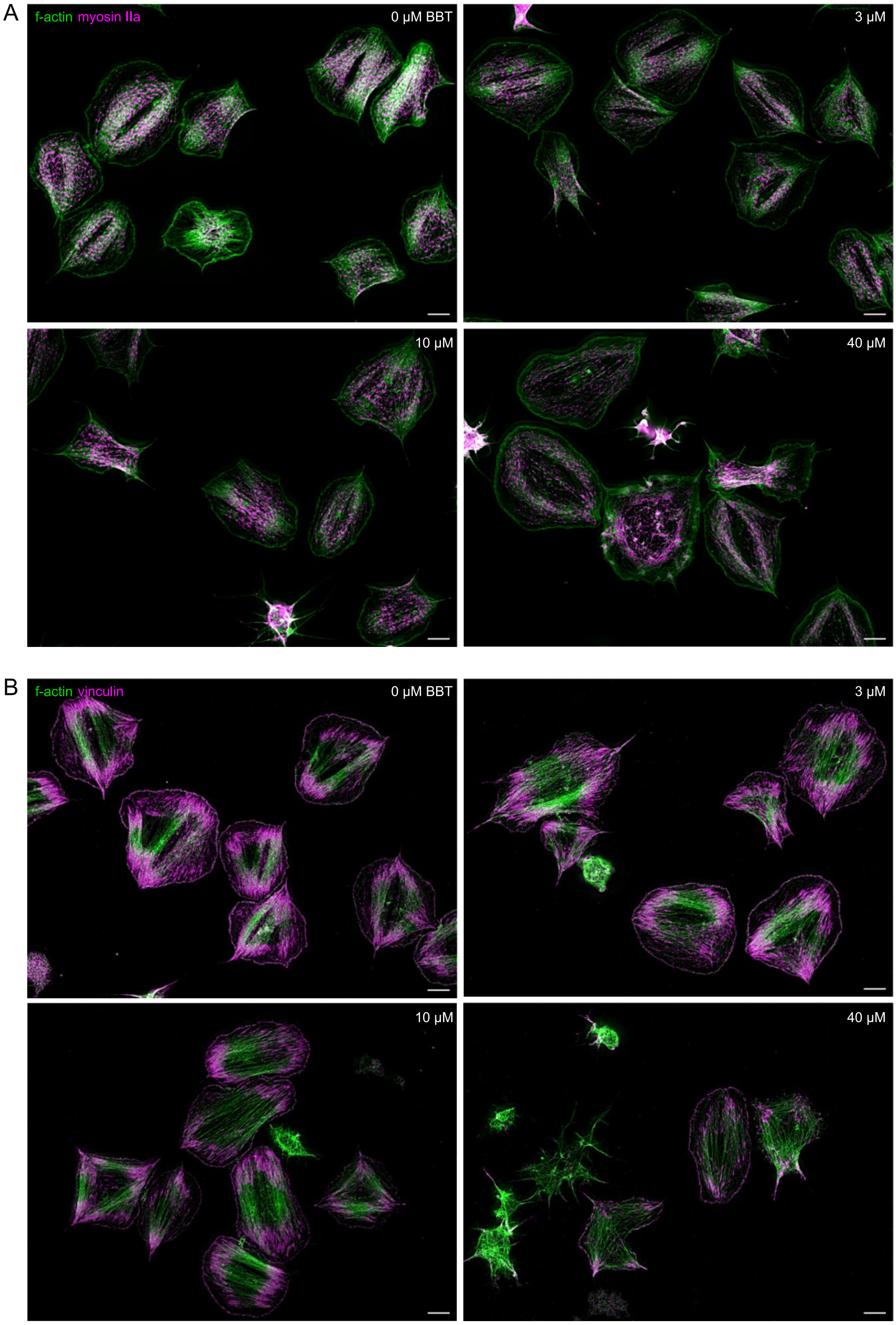

Supplementary Figure S3: τSTED microscopy overview images of washed human platelets spread on fibrinogen for 60 minutes in the presence of 0, 3, 10 or 40 µM para-amino BBT. Fixed samples were stained for f-actin (green) and
A) myosin IIa (magenta) or
B) vinculin (magenta).
Scale bars: 2 µm.

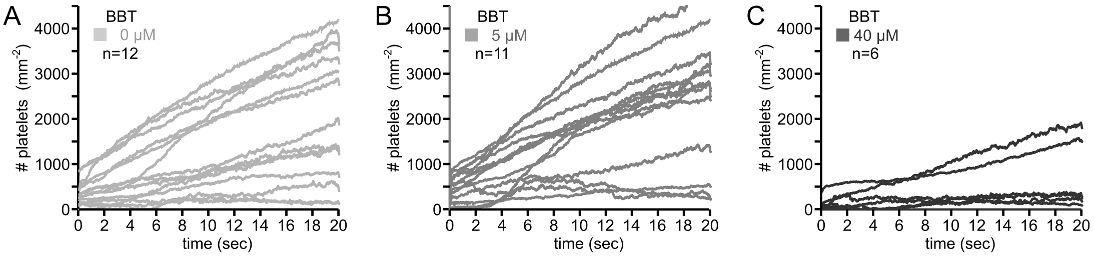

Supplementary Figure S4: Initial platelet:vWF interactions under arterial shear in the absence (A) or presence of 5 µM para-amino BBT (B) or 40 µM para-amino BBT (C). Shown is the number of platelets per square millimeter over time. Single platelets were detected from fluorescence images of vWF-coated channel bottoms when $DiOC_6$-stained whole blood was perfused through the flow channel at a shear rate of 1500 s^{-1}. Each trace shows an independent experiment.

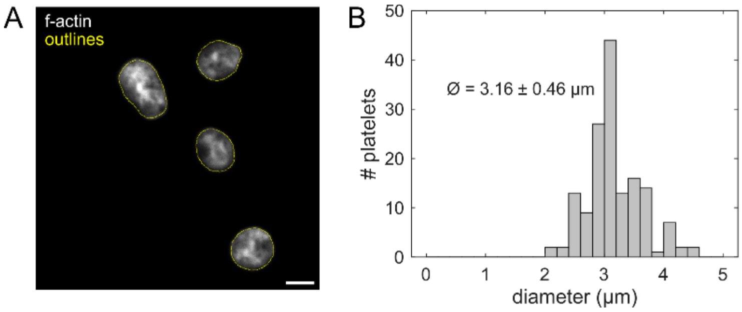

Supplementary Figure S5: Diameter of resting platelets.
A) τSTED images of f-actin in resting platelets. Note that the f-actin staining is dispersed throughout the platelets. Resting platelets were fixed in solution by 1% glutaraldehyde, spun down onto fibrinogen-coated coverslips, permeabilized, stained with phalloidin-Star635P, mounted, and imaged by τSTED. Cell outlines (yellow) were determined by intensity thresholding. Scale bar: 2 µm.
B) Distribution of single platelet diameters. The diameter was calculated from the area A enclosed by the outline using $d = 2\sqrt{A/\pi}$.

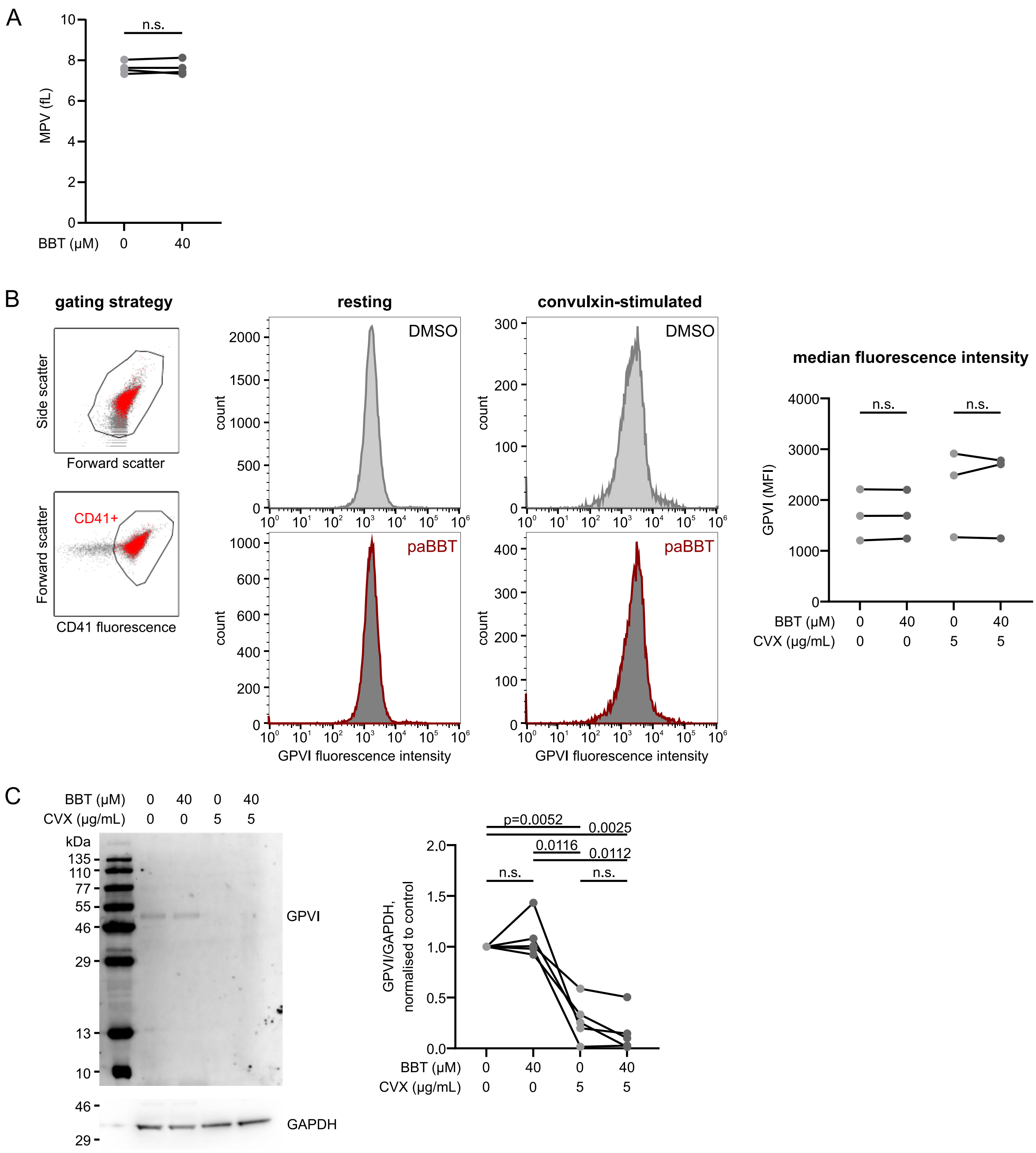

Supplementary Figure S6: Myosin IIA inhibition by BBT has no effect on mean platelet volume (MPV), GPVI surface expression levels, and GPVI shedding.
(A) MPV measurements of resting platelets incubated at 30°C for 10 min with 40 µM para-amino BBT or vehicle control (DMSO). Statistical comparisons (n=4) were made using a paired Student's t-test.
(B) Surface expression levels of GPVI in resting platelets after incubation at 30°C for 10 min with 40 µM para-amino BBT or vehicle control (DMSO), as well as in suspended platelets after stimulation with convulxin (5 µg/mL) at room temperature for 60 min. Flow cytometric data was gated based on forward/side scatter and CD41 intensity (left), and the median fluorescence intensity (MFI) of GPVI was measured (right) from GPVI intensity of the platelet population (middle, representative of n=3 independent experiments). Statistical comparisons were made using a paired Student's t-test.
(C) Assessment of GPVI shedding. After stimulation of platelets with convulxin (5 µg/mL) at room temperature for 60 min, platelets were pelleted, lysed, and blotted for GPVI and GAPDH. The used anti-GPVI antibody recognizes the extracellular (shed) portion of GPVI. Left: representative western blot showing a marked reduction of band intensity in convulxin-treated samples due to shedding. Right: quantification and statistical comparison by one-way ANOVA for paired data using the Geisser-Greenhouse correction (n=5).

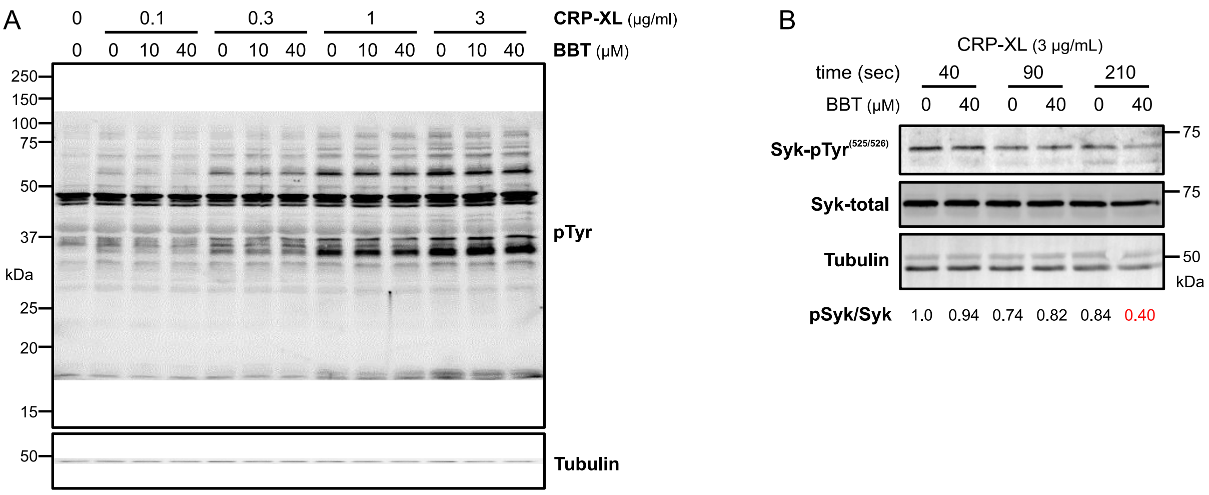

Supplementary Figure S7: GPVI-stimulated changes in overall tyrosine phosphorylation or Syk phosphorylation at different times. (A) Washed platelets were pre-incubated with 0, 10 or 40 µM para-amino BBT for 10 minutes before stimulation with 0, 0.3, 1 or 3 µg/ml CRP-XL or vehicle for 90 seconds. (B) Washed platelets were pre-incubated with 0 or 40 µM para-amino BBT for 10 minutes before stimulation with 3 µg/ml CRP-XL or vehicle for 40, 90 or 210 seconds.

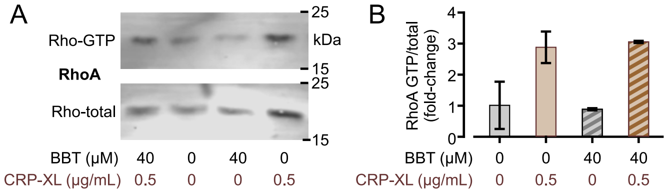

Supplementary Figure S8: GPVI-stimulated RhoA activity in non-adherent platelets is not affected by myosin inhibition. Washed platelets were pre-incubated with 0 or 40 µM para-amino BBT for 10 minutes before stimulation with 0.5 µg/ml CRP-XL or vehicle for 3 minutes. GTP-bound Rho GTPases were pulled down using GST-Rhotekin-RBD.

A) Western Blots of Rho-GTP and total Rho visualized by mouse anti-RhoA antibodies followed by IRDye680-anti-mouse and detected using a LICOR-Odyssey scanner and quantified using ImageStudioLite software (LICOR).

B) Quantification of GTP-bound to total RhoA from Western blots. para-amino BBT did not cause any RhoA activation in the vehicle control, nor did it affect CRP-induced activation of RhoA (one way ANOVA with Bonferroni correction). Shown are mean and s.e.m. of =2 technical replicates.

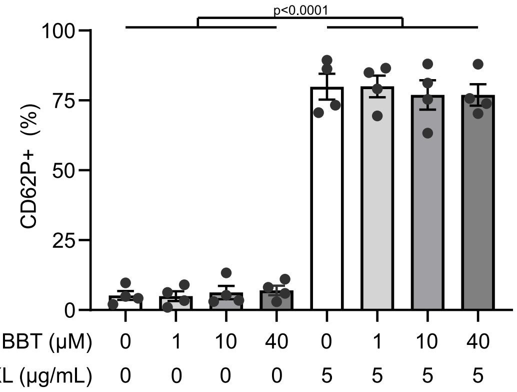

Supplementary Figure S9: GPVI-stimulated α-granule secretion in solution is not affected by myosin inhibition. Washed platelets were pre-incubated with 0, 1, 10 or 40 μM para-amino blebbistatin (para-amino BBT) for 10 minutes before stimulation with 5 μg/ml CRP or vehicle. P-selectin was stained using anti-CD62P immunostaining and assessed by flow-cytometry. Shown are individual values, mean and s.e.m. para-amino BBT did not cause any pre-activation in the vehicle control, nor did it affect P-selectin expression on CRP-stimulated platelets significantly. Statistical significance was tested by one way ANOVA with Bonferroni correction; only p-values <0.05 are indicated.

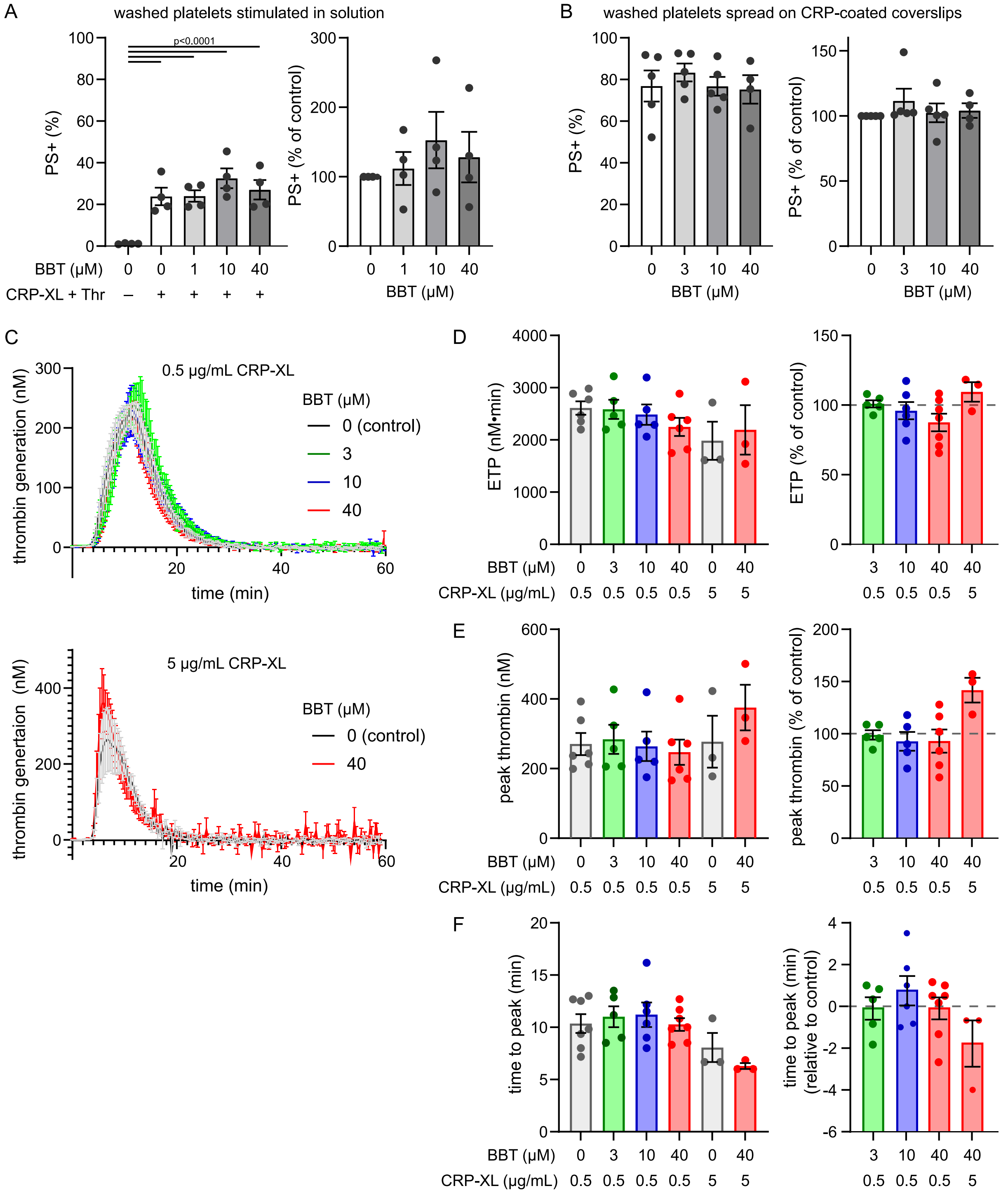

Supplementary Figure S10: GPVI-stimulated procoagulant platelet formation is not affected by myosin inhibition. A) PS exposure of washed platelets stimulated by 0.5 μg/ml CRP-XL and 1 U/ml thrombin for 30 minutes in solution in the presence of 0, 1, 10 or 40 μM para-amino blebbistatin (para-amino BBT). PS exposure was measured by AnnexinV binding using flow cytometry in 4 independent experiments.

B) PS exposure of washed platelets spread on CRP-XL coated coverslips for 30 minutes in the presence of 0, 1, 10 or 40 μM para-amino BBT. PS exposure was visualized by AnnexinV binding and PS+ platelets were counted by 3 independent researchers in a blinded fashion. Data from 4-5 independent experiments with (mean±std) 142±93 cells per condition.

C) Thrombograms of platelet-based thrombin generation assay (TGA) showing the mean (line) and standard error (error bars) of 3-5 independent experiments per condition measured in duplicate. Platelet-rich plasma containing 40 μM RUC-2 (or 10 μg/ml eptifibatide) to block aggregate formation as well as 0, 3, 10 or 40 μM para-amino BBT was stimulated with 0.5 μg/ml (upper panel) or 5 μg/ml (lower panel) of CRP-XL for 10 minutes at 37°C before starting the TGA. Thrombin activity was measured by a fluorogenic substrate over 60 minutes.

D) Endogenous thrombin potential (=area under the curve) derived from thrombograms in C.

E) Peak thrombin (=maximum value) derived from thrombograms in C.
F) Time to peak derived from thrombograms in C.
Shown are individual data points, mean and s.e.m. of raw values (left) and of experiments normalized to the matching control (right) which partially accounts for donor-to-donor variability. Statistical significance was tested by one-way ANOVA with Bonferroni correction for multiple comparisons; only p-values <0.05 are indicated.
